# Prevalence and associated factors of sexually transmitted infections among unmarried undergraduate university students

**DOI:** 10.1371/journal.pone.0358430

**Published:** 2026-09-24

**Authors:** Mary Aku Ogum, Joseph Kwame Mintah, Prosper Narteh Ogum, Jacob Owusu Sarfo, Thomas Hormenu, Edward Wilson Ansah

**Affiliations:** 1 Department of Health, Physical Education and Recreation, University of Cape Coast, Cape Coast, Ghana; 2 Cardiometabolic Epidemiology Research Laboratory, University of Cape Coast, Cape Coast, Ghana; Amrita Vishwa Vidyapeetham - Amritapuri Campus, INDIA

## Abstract

**Background:**

Many young university students are sexually active, but unable to properly handle the responsibilities that come with having sexual intercourse. This could lead to higher rates of sexually transmitted infections. Such behaviours may be because many of these students are becoming independent for the first time as they enter university. Therefore, the study aimed to investigate the prevalence and factors associated with the rate of sexually transmitted infections among a cohort of young unmarried university students in a developing country, Ghana.

**Method:**

This survey used a convenience sampling method to recruit a large number of undergraduate university students from Ghana. A modified Core Expanded Questions of the Global School-based Student Health Survey [GSHS] questionnaire was used for data collection on sexual behaviours that contribute to HIV/STI and unintended pregnancies. Frequencies, percentages, chi-square, and binary logistic regression were used to analyse the data.

**Results:**

The results revealed that out of the 10,896 students, 5,448 (50%) were male and 5,448 (50%) were female. Overall, 4,124 (37.8%) students were sexually active, whereas 6,772 (62.2%) were not. Among the sexually active unmarried students, 1,506 (36.5%) reported having ever been infected with at least one STI, mostly gonorrhea and syphilis. Furthermore, females (n = 835:62.2%), students aged 20–24 years (n = 838;62.4%), level 200s (n = 443;33.0%), and level 300s (juniors) (440;32.8%), Christians (n = 1007;75%), non-resident students (n = 798;59.0%), those with low socioeconomic status (912;67.9) and those who did not use condom at their last sexual intercourse (897;66.8%) were more likely to be infected with at least an STI. Students aged 20–24 years (AOR = 3.620, 95%CI = 2.435–5.382), those aged 25 years and above (AOR = 1.457, 95%CI = 1.237–1.716), and those at level 300 (juniors)(AOR = 1.038, 95%C.785-1.373) were more likely to suffer from STI. Moreover, Muslims students (AOR = 1.88, 95%CI = 1.312–2.703), students from other religions (AOR = 1.390, 95%CI = .939-2.056), those who engaged in anal sex (AOR = 1.351, 95%CI = 1.123–1.624), those who did not use condom during their last sexual encounters (AOR = 1.355, 95%CI = 1.03–1.665) and those whose friends were sexually active (AOR = 3.713, 95%CI = 2.132–6.468) were more likely to report infection with STIs.

**Conclusion:**

There is a high prevalence of STIs among unmarried young university students in Ghana because of an increase in risky sexual behaviours. Unfortunately, this is making the fight against HIV/AIDS and other deadly STIs complicated. Thus, institution-based health education and promotion programmes are required to lower the risk of STIs, including HIV/AIDS, among the students. These findings pose challenges to achieving SDGs 3 and 17, which highlight the need for worldwide collaboration and accelerated efforts to curb STIs by 2030.

## Background

The global HIV/AIDS epidemic is still a public health concern, especially when young people are getting sexually active early in life [1]. Unfortunately, this early sexual initiation is not marching with the ability of adolescents and young adults for self-protection, leading to an increase in HIV/AIDS and other sexually transmitted infections (STIs). STIs are a group of infections or clinical syndromes caused by pathogens acquired and transmitted from one person to another, mainly through unprotected vagina, anal and oral sexual intercourse [2]. STIs may contribute substantially to morbidity and mortality globally, particularly among young people, due to a range of behavioural and structural risk factors [2]. STIs are part of the common acute health conditions that have long-term complications, including psychological consequences and even death [3,4]. The complications from STIs may include pelvic inflammatory disease, infertility, ectopic pregnancies, loss of pregnancy and stillbirth, cervical cancer, and preterm labour [5]. In men, undiagnosed and untreated STIs may lead to epididymo-orchitis and subsequent subfertility [5].

Even though there are about 30 bacterial, viral and parasitic pathogens that transmit via sexual infections, eight are linked to the greatest incidence of STIs. These eight include chlamydia, trichomoniasis, gonorrhoea, syphilis, hepatitis B virus, herpes simplex virus, human papillomavirus (HPV) and human immunodeficiency virus (HIV) [6]. Among the STIs mentioned, the first four are curable with antibiotic treatment because they are caused by either bacteria or parasites [7]. However, the latter four are not curable, but prompt diagnosis and treatment can help alleviate symptoms and prevent long-term health complications [8,9].

The global prevalence of STIs remains high with about one million daily infections and half a billion people fall sick from one of the four curable STIs [3,7,10]. Estimate indicated that each year, 376 million new cases of the four curable STIs occur among people aged 15–49 years [2,4,10–12]. Meanwhile, 536 million people are living with the incurable herpes simplex virus type 2 infection and the HIV [10], but between 75% and 85% of such infections are estimated to occur in developing countries. The evidence is that infection with STIs increases the chance of HIV transmission and infection risk by two to three times [12–14]. Unfortunately, STIs, including HIV/AIDS are a big public health concern in sub-Saharan Africa [SSA] [6] because they account for 17% of the overall burden of disease and are one of the leading causes of sickness among women of reproductive age [15].

Beyond the health burdens, STIs have serious social and economic consequences. For instance, Marwan et al. [16] documented that many STIs cases are hidden and are not captured in health or other data because many people feel stigmatised when trying to seek medical care. Additionally, United States Centers for Disease Control and Prevention’s (CDC) analysis of the cost of treatment and prevention of STIs indicated that STIs costs society nearly $16 billion each year [17,18]. Specifically, about $13.7 billion of the costs were attributed to sexually acquired HIV infections, $755 million attributed to HPV infections, more than $1 billion attributed to chlamydia, gonorrhea and syphilis combined. Moreover, about 60% of the costs from chlamydia, gonorrhea and syphilis were among young people aged 15–24 years. The observation is that, this is the age category for university students [17,19,20].

Since STIs cause serious health and social menace to communities, World Health Organisation (WHO) proposed and adopted an approach in 2016 to end STIs while improving individual’s health, men’s and women’s sexual health, and the well-being of all people [3]. Evidence suggests that STIs (and their complications) are among the top five disease groups for which adults seek medical attention in low-and middle-income countries (LMICs) [3]. So, to achieve WHO’s targets, research is needed in all populations to reveal the global burden of STIs on the youth in these less-resourced nations [3]. However, studies in Ghana on STIs have focused mainly on women [21–25] and adolescents in second cycle institutions [26,27], with none so for targeting young people in tertiary institutions. Since the absence of evidence on the prevalence and associated factors of STIs among this well-defined population of young people can negatively affect the success of any effective interventions aimed at curbing the menace, it is necessary to get baseline evidence among undergraduate students in Ghana. Such studies are important to develop interventions, including public health campaigns, toward the attainment of the United Nations’ Sustainable Development Goals 3. Moreover, such evidence is needed for the attainment of the global health sector strategy on STIs 2016–2021 (thus, promoting healthy lives and ending STIs epidemics as a major public health concern worldwide by 2030) [12]. Hence, this study assesses the prevalence and associated factors of STIs among unmarried university students in Ghana.

### Theoretical framework: Social ecological model (SEM)

The Social Ecological Model (SEM) provides the theoretical foundation for this study by explaining how multiple levels of influence interact to shape health behaviours and outcomes, as well as the risk of sexually transmitted infections (STIs) among unmarried undergraduate university students [28]. The model acknowledges the interpersonal dimension of behaviour and identifies that health behaviours are influenced not only by individual characteristics but also by interpersonal relationships, organisational contexts, community environments, and broader societal factors [29,30]. This multilevel perspective is particularly important when considering STI prevention, as sexual behaviours do not occur in isolation, but are influenced by a variety of personal, social, and environmental factors rather than by individual choices alone [31].

In this study, the SEM provides a framework for understanding how factors at different levels may influence the occurrence of STIs. At the individual level, characteristics such as age, gender, knowledge of STIs, sexual history, condom use, substance use during sexual activity, and the number of sexual partners may affect students’ sexual decision-making and vulnerability to STIs [32,33]. At the interpersonal level, peer influence, the number of sexually active friends, and parental communication may shape sexual behaviours through social support, behavioural norms, and communication about sexual health [34,35]. Organisational and community influences, together with access to university sexual health services, sexual health education, cultural beliefs, religious norms, and stigma surrounding STIs, may further affect students’ ability to engage in preventive behaviours and seek timely testing and treatment [30,36].

The use of the SEM in this study recognises that the prevalence of STIs among unmarried undergraduate students is not merely caused by individual risk behaviours, but by a combination of multiple factors. Although the present study focuses on individual- and interpersonal-level determinants, the SEM offers a broader framework for understanding the findings. It also underscores the importance of multilevel interventions to enhance sexual health education, promote positive peer and family support, improve access to sexual health services at the university, and mitigate social and cultural barriers to STI prevention [30,34,36].

## Methods

### Design and participants

This cross-sectional survey assessed the prevalence and correlates of STIs among a total of 10,896 unmarried undergraduate university students in Ghana. These university students are from diverse academic disciplines. They were selected based on their willingness to participate in the study. The age of the students varies from below 20 to above 25 years, who are from different socio-economic backgrounds. These students were chosen because of their unique experiences and views as university students that provide valuable insights into STIs and factors that influence their contraction and transmission.

**Procedure** The sample size was determined based on the guidelines provided by Babbie [37], which state that sample size should be determined by study purpose, precision of estimates desired, characteristics of study population, and planned analytical procedure. Although statistical sample size formulas provide the minimum number of participants required to generate population estimates, a larger sample was considered appropriate for this study to improve the precision and reliability of estimates, enhance statistical power, and provide sufficient numbers for subgroup analyses and multivariable modelling [38,39]. Additionally, given the sensitive nature of the study, which involved questions regarding sexual behaviours and sexually transmitted infections, a larger sample was deemed necessary to account for potential underreporting and non-disclosure of sensitive information [40]. Therefore, a sample size of 10,896 regular undergraduate students, representing approximately 60% of the total undergraduate population, was collected and used for the study.

The 10,896 sample size was stratified into four levels (100, 200, 300, and 400), representing 2,724 students for each level. Each of the levels was then divided equally into males and females, consisting of 1,362 each. Again, the males and females for each level were evenly divided into residential and non-residential, comprising 681 students each. Then, an accidental sampling technique was used to select participants for the study. This sampling technique was chosen because of the sensitive nature of the study, which does not easily permit participants to consent to take part. Also, this sampling technique was deemed appropriate to get access to the participants because of the widespread nature of students on campus. The levels 100, 200, 300 and 400, used to categorise undergraduates in this study, are analogous to freshmen, sophomores, juniors and seniors in the United States.

The sample for the study was all regular, unmarried undergraduate students who were officially enrolled and actively attending lectures during the period of data collection [40]. These students formed the target population because they represented the group most consistently exposed to all kinds of lifestyles, which could predispose them to unprotected sexual activities that can increase their chances of acquiring STIs. The inclusion criteria comprised only regular, unmarried undergraduate students who were willing to participate and provided informed consent. However, students who are married, postgraduate, sandwich,students were excluded from the study because their academic structure, workload, and lifestyle differed significantly from those of undergraduates, which could introduce variability in stress levels and physical activity patterns [41].

The questionnaire for data collection was anonymous and administered by the researcher with the help of 12 trained research assistants who formed four groups of three individuals. Canopies were erected at the forecourts of halls, hostels and the main library of the university, where it was easy to have access to a large group of students. The canopies were partitioned to provide privacy and comfort for the participants when filling out the questionnaire. Three research assistants were assigned to each location during the questionnaire administration. Students who walked passed or walked to the erected canopies were invited to take part in the study. Those who consented to take part in the study signed an informed consent form after they had been assured of anonymity and confidentiality of their data. Those who consented were ushered individually into any of the cubicles where a table and a chair had been provided for them to answer the questionnaire. Participants were asked to drop the completed questionnaire in a box placed at the corner of the cubicle.

Students were escorted individually into the cubicles and left alone to answer the questionnaire, so that no other students or research assistants could see their responses to the items. This was done strictly to ensure the confidentiality of students’ responses to the items. Students spent about 15 minutes filling out the instrument. In accordance with the Declaration of Helsinki on ethical clearance for human subjects, ethical consideration (UCCIRB/CES/2019/46) of the study protocol was approved by the University of Cape Coast Institutional Review Board, and data collection was between January and February 2020.

### Instrument

The Core Expanded Questions of the Global School-based Student Health Survey [GSHS] was adapted for the data collection [G-YRBS] [42,43]. The GSHS questions examine sexual behaviours of students that may contribute to HIV/STI, unintended pregnancies, and abortions [44–46]. Ten relevant items were selected from the GSHS to form our questionnaire for this study. In addition, we collected students’ socio-demographic information on age, gender, age at first sexual intercourse, condom use, multiple sexual partnerships, sexual intercourse preferences, and use of psychoactive drugs. The instrument was pretested on 350 regular unmarried undergraduate students from a sister university in Ghana. This university was chosen because the students have similar characteristics to their counterparts in the university where the study was conducted. The multi-point scale items yielded an internal consistency reliability coefficient of.982 [36]. Conversely, the multiple-choice items also yielded a reliability coefficient of.892 [36].

### Measures

Self-reported STI status among the students was the dependent variable (DV), which was framed as ever suffered from or currently being diagnosed with an STI (Table 1). Before responding to the questions on STI items, participants were provided with a brief explanation of what sexually transmitted infections (STIs) are in the questionnaires: “STIs are infections that are transmitted through sexual contact.” Participants were then asked whether they had ever suffered from or were currently diagnosed with an STI by a healthcare professional. They responded to this question with “Yes” or “No”, which were categorised as Yes = 1 for those who had ever suffered from or were currently diagnosed with an STI, and No = 2 for those who had never suffered from an STI. In a follow-up question, participants were presented with a list of common sexually transmitted infections and were asked to indicate the specific infection(s) for which they had previously or currently received a diagnosis. We considered STIs such as gonorrhea, syphilis, chlamydia, genital herpes, trichomoniasis, HIV, and genital warts. Moreover, we used gender (male and female), age (≤20 years, 20–24 years, and 25 years and above), age at first sexual intercourse (below 18 years and 18 years and above), condom use during sex (yes, no), academic level (100, 200, 300, and 400, or freshman, sophomore, junior, and senior), residential status (residential and non-residential), sexual intercourse preference (vaginal and anal), perception of sexual behaviour (safe and risky), socioeconomic status (low and high socioeconomic status), religious affiliation (Christian, Muslim, and other), parental communication (difficult and easy), peer influence (having sexually active friends and non-sexually active friends), drug and substance use (sex-enhancing drugs, marijuana, alcohol, or other psychoactive drugs), and number of current sexual partners (one and more than one) as the independent variables (IVs). Additionally, the construct sexual behaviour was operationalised using self-reported measures of behaviours known to influence STI risk. This included age, university standing (academic level), religious affiliation, sexual preference, condom use, number of sexually active friends, and current number of sexual partners, age at first sexual intercourse, condom use, drug use before or during sex, and other sexual practices that were measured in the questionnaire.

**Table 1:** Definition of explanatory and measurement coding of variables.

Gender		1 = Male
		2 = Female
Chronological age	How old are you?	1 = less than 20 years 2 = 20–24 years 3 = 25 years and above
Age at first sex	How old were you when you first had sexual intercourse?	1= below 18 years 2= above 18 years
Academic level	Which level are you?	1 = level 100 2 = level 200 3 = level 300 4 = level 400
Residential status	Are you a residential or non-residential student?	1 = residential 2 = non-residential
Religious affiliation	What is your present religion?	1 = Christian 2 = Muslim 3 = Others
Parental communication	How easy is it for you to talk to the person you stay with about sex?	1 = difficult 2 = easy
Sexual preference	Which of the following do you practice?	1 = vagina penile penetration 2 = anal penile penetration
Sexual behaviour (It is made up of composite score from the various variables in the middle with asterisk)	*Did you use condom at your last sexual intercourse? *Which do you practice? *How many sexual partners do you currently have? *Which of these drugs have you ever used before sex? *How often do you use selected drugs?	1 = safe sexual behaviour 2 = risky sexual behaviour
Condom use	Did you use condom at your last sexual intercourse?	1 = yes 2 = no
Number of sexually active friends	How many of your friends are sexually active?	1 = none of them 2 = all of them 3 = most of them 4 = some of them 5 = few of them
Current number of sexual partners	How many sexual partners do you currently have?	1 = none 2 = one person 3 = 2 persons 4 = 3 persons 5 = more than three persons
Drug use	How often do you use drugs before sexual intercourse?	1 = never 2 = always 2 = once a while

### Analysis

Data was managed using the Statistical Package for Social Sciences (SPSS) software Version 20.0. We used frequency and percentage analysis to explore the socio-demographic variables of the students. Moreover, Pearson Chi-square was used to determine the association between having STIs and the IVs. Additionally, a binary logistic regression model was built to test the predictive power of the IVs on infection of STI among the students, at 95% confidence interval (CI) and p < 0.05.

## Results

From Table 2, the prevalence of STIs among regular unmarried undergraduate students stood at 36.5% (1506), with more females, (62.2%; n = 835) suffering the infection. Also, 62.4% (838) of undergraduates between ages 20 and 24, 33.0%(443) of those in level 200 and 32.8%440) of level 300 suffer the infections. Likewise, 75.0%(1007) of the Christians and 59.0%(798) of the non-residential students suffer the infections. Again, 67.9%(912) of the students from a low socioeconomic background and 66.8% (897) of those who do not use a condom during their last sexual intercourse suffer STIs.

**Table 2:** Prevalence of the various STIs.

STIs ever suffered/currently suffering	Yes		No	
	F	%	F	%
Gonorrhea	805	19.5	3319	80.5
Syphilis	305	7.4	3819	92.6
Trichonomia	71	1.7	4053	98.3
Chlamydia	125	3.0	3999	97.0
Genital herpes	96	2.3	4028	97.7
HIV/AIDS	64	1.6	4060	98.4
Genital warts	40	1.0	4084	99.0

*The crude prevalence and specific STIs prevalence among unmarried undergraduate students in Ghana*

### Chi-Square analysis of self-reported STIs

From Table 3, the Chi-Square test shows that chronological age (χ^2^ = 82.933, p < 0.000), academic level (χ^2^ = 99.425, p < 0.000), residential status (χ^2^ = 15.237, p < 0.000), religious affiliation (χ^2^ = 34.290, p < 0.000), socioeconomic status (χ^2^ = .923, p < 0.033), sexual preference (χ^2^ = 41.121, p < 0.000), sexual behaviour (χ^2^ = 82.616, p < 0.000), condom use at last sexual intercourse (χ^2^ = 9.186, p < 0.002), number of sexually active friends (χ^2^ = 23.395, p < 0.000), number of current sexual partners (χ^2^ = 148.437, p < 0.000), and frequency of drug use for sex (χ^2^ = 441.739, p < 0.000) were significantly associated with STIs among unmarried university students in Ghana (see Table 3).

**Table 3:** Bivariate association of selected factors and contracting of STIs by unmarried regular undergraduate university students.

		STIs (4124)				
		**Yes** **(%)**	**No** **(%)**	**Chi-square (χ**^**2**^)	**Sig**	**Φc**
Age	Less than 20 yrs 20–24 years 25 & above yrs	42(13.8%) 831(30.9%) 456(40.3%)	262(86.2%) 1857(69.1%) 676(59.7%)	82.933	.000	.142
Academic level	Level 100 Level 200 Level 300 Level 400	148(20.9%) 441(41.7%) 433(34.8%) 307(27.6%)	559(79.1%) 617(58.3%) 812(65.2%) 807(72.4%)	99.425	.000	.155
Residential status	Residential Non residential	536(29.1%) 793(34.8%)	1308(70.9%) 1487(65.2%)	15.237	.000	−.061
Religious affiliation	Christians Muslims Traditional	993(30.2%) 263(38.7%) 73(46.8%)	22959(69.8%) 417(61.3%) 83(53.2%)	34.290	.000	.091
Socioeconomic status	Low socio- economic status High socio- economic status	903(32.7%) 426(31.2%)	1857(67.3%) 938(68.8%)	.923	.337	.015
Sexual preference	Vagina sex Anal sex	1041(30.2%) 288(42.8%)	2410(69.8%) 385(57.2%)	41.121	.000	−.100
						
Sexual behaviour	Safe sexual behaviour Risky sexual behavior	171(19.5%) 1158(35.7%)	706(80.5%) 2089(64.3%)	82.616	.000	−.142
Condom usage	Yes No	439(29.3%) 890(33.9%)	1059(70.7%) 1736(66.1%)	9.186	.002	−.047
Number of sexually active friends	All of them Most of them Some of them Few of them None of them	169(30.3%) 476(36.0%) 358(29.9%) 306(32.8%) 20(17.7%)	389(69.7%) 848(64.0%) 839(70.1%) 626(67.2%) 93(82.3%)	23.395	.000	.075
Current number of sexual partners	1 person 2 persons 3 persons >3 persons None	627(27.9%) 388(39.1%) 144(41.9%) 143(49.5%) 27(10.6%)	1617(72.1%) 605(60.9%) 200(58.1%) 146(50.5%) 227(89.4%)	148.437	.000	.190
Frequency of drug use during sex.	Always Once a while Never	224(55.2%) 569(51.1%) 536(20.6%)	182(44.8%) 544(48.9%) 2069(79.4%)	441.739	.000	.327

### Factors predicting STIs among unmarried regular undergraduate university students

The results in Table 4 show that undergraduates aged 20−24 (AOR = 3.620, 95%CI = 2.435–5.382) and 25 years and above (AOR = 1.457, 95%CI = 1.237–1.716) were more likely to suffer STIs relative to those less than 20 years. Again, level 300 students (AOR = 1.038, 95%C.785-1.373) were more likely, while those in 200 (AOR = .458, 95%CI = .371−.565) and 400 (AOR = .883, 95%CI = .725-1.076) were less likely to suffer STIs compared with those in level 100. Likewise, students who engaged in anal sex were more likely than those who engaged in vaginal-penile sex (AOR = 1.351, 95%CI = 1.123–1.624), and those who had sexual intercourse without a condom (AOR = 1.355, 95%CI = 1.03–1.665) were more likely to suffer STIs. Moreover, students who believed all their friends are sexually active were more likely to suffer STIs (AOR = 3.713, 95%CI = 2.132–6.468) compare with those who believed most (AOR = .897, 95%CI = .692-1.162), some (AOR = .975, 95%CI = .761-1.248) and a few of their friends (AOR = .899, 95%CI = .708-1.141) are sexually active. Furthermore, students who had 1−3 (AOR = .305, 95%CI = .199−.468) and more than 3 sexual partners (AOR = .258, 95%CI = .166−.401) were less likely to suffer STIs. Lastly, students who use drugs for sex always (AOR = .230, 95%CI = .177−.299) and once while (AOR = .264, 95%CI = .219−.318) were less likely to suffer STIs. Additionally, the Hosmer and Lemessow test (Goodness of Fit), χ^2^ (8) = 22.829, p = 0.004, indicated that the model did not fit the data optimally. Table 4 presents the results on factors influencing STIs among unmarried undergraduate students.

**Table 4:** Factors associated with STIs among unmarried regular undergraduate university students.

Variables	B	X^2^	Sig.	Adjusted Odds Ratio (AOR)	95% Confidence interval for odds ratio	
					**Lower**	**Upper**
*Age*						
Less than 20 years						
20–24 years	1.287	40.455	.001	3.620	2.435	5.382
25 years and above	.376	20.250	.001	1.457	1.237	1.716
*Academic level*						
Level 100						
Level 200	.037	.069	.793	1.038	.785	1.373
Level 300	−.781	52.904	.001	.458	.371	.565
Level 400	−.124	1.516	.218	.883	.725	1.076
*Residential status*						
Residential						
Non-residential	−.084	1.040	.308	.919	.782	1.081
*Religious affiliation*						
Christians						
Muslims	.633	11.773	.001	1.883	1.312	2.703
Other religions	.329	2.710	.100	1.390	.939	2.056
*Sexual preference*						
Vagina penile penetration						
Anal penile penetration	.301	10.220	.001	1.351	1.123	1.624
*Sexual behaviour*						
Safe sexual behavior						
Risky sexual behavior	−.173	1.430	.232	.841	.633	1.117
*Condom usage*						
Yes						
No	.304	8.362	.004	1.355	1.103	1.665
*No. of friends sexually active*						
None of them						
All of them	1.312	21.473	.001	3.713	2.132	6.468
Most of them	−.109	.679	.410	.897	.692	1.162
Some of them	−.026	.042	.838	.975	.761	1.248
Few of them	−.107	.771	.380	.899	.708	1.141
*Current no. of sexual partners*						
None						
1 person	−1.188	29.494	.001	.305	.199	.468
2 persons	−1.354	36.481	.001	.258	.166	.401
3 persons	−1.378	31.957	.001	.252	.156	.407
More than 3 persons	−1.641	42.906	.001	.194	.119	.317
*Freq. of drug use during sex*						
Never						
Always	−1.468	121.443	.001	.230	.177	.299
Once a while	−1.333	195.175	.001	.264	.219	.318
Constant	1.867	35.851	.001	6.469		

Hosmer and Lemeshow test (goodness of fit), χ^2^ (8) = 22.829, p=0.004

### Summary of findings

The crude prevalence of STIs among the students stood at 36.5%, with more females (62.2%) suffering from the diseases. Also, 62.4% of the students between the ages 20 and 24 and 33.0% in level 200 and 32.8% in level 300 suffer from the infections. Likewise, 75% of the Christians and 59.0% of non-residential students suffer STIs, with the majority (67.9%) from economically disadvantaged homes and almost two-thirds (66.8%) of those who do not use condom during their last sexual intercourse suffering from STIs.

The finding is that students aged 20–24 and those 25 years and above were more likely to suffer STIs relative to those less than 20 years. Again, level 300 students were more likely, while those in 200 and 400 were less likely to suffer STIs compared with those in level 100. Likewise, students who engaged in anal sex, had sexual intercourse without the use of condom, and those who believed all their friends are sexually active were more likely to suffer STIs. Moreover, students who have 1–3, those who currently have more than 3 sexual partners and those who use drugs always and once a while for sex were less likely to suffer STIs.

## Discussion

### Prevalence of STIs among unmarried undergraduate students in Ghana

There is a high (36.5%) prevalence of STIs among undergraduate students in Ghana. These findings may point to increased vulnerability to risky or potentially exploitative sexual situations among students in this group. A similia prevalence of STIs was reported among adolescent girls and young women in SSA [34]. However, the prevalence in the current finding is higher than those found in studies conducted at Mekelle in Northern Ethiopia (21.3%) among young females [34], another (20.6%) from Brazil [33] and among Wolaita Sodo university students (19.5%) in southern Ethiopia [35]. However, other studies recorded much lower rate of STIs than that found in the current study, like 18.2% found among university of Gondar students in Northwest Ethiopia [20] and 14.3% among the students in Southwest Ethiopia [36]. Perhaps, these observed differences or variations in reported prevalence rates across studies may be partly due to the sampling approach used and also partly because we collected our data and studied only university students who have not yet married. Moreover, this data was collected using accidental sampling method in recruiting the students. We are of the view that this category of students have higher propensity to engage in vice sexual exploitation, especially when some of them had never had such freedom as they are experiencing on the university campus currently. Additionally, applying accidental or voluntary sampling method [36], it is likely that we recruited students who thought they had issues concerning STIs, thus, were volunteering to participate in this. Thus, the prevalence of STIs found in this study calls for implementing strategies and programmes that could increase STI testing, treatment and prevention among the students. The issue is that such measures contribute to achieving the global health strategy for STIs, which envisages a near future and world where each person everywhere has a free and easy access to STI prevention and treatment services [37].

### Correlates of STIs among unmarried undergraduate students in Ghana

The finding further indicated that undergraduates aged 20–24 years and those 25 years and above were more likely to suffer STIs compared to their counterparts who are less than 20 years. Thus, the likelihood of the students getting infection with STIs increases with age. Our findings align with the previous studies conducted in Malawi [38], Central African Republic, Eswatini and Ghana [39], Ghana and Kenya [40] and Ghana [41], which recorded that, women, undergraduates aged 20–24 years and above, young people and adolescents are more likely to engage in risky sexual behaviours such as having multiple sexual relationships and having sex without condoms predisposing them to higher odds of contracting STIs. The findings from other prior studies are that youths including young women age 20–24 have higher risk of developing STIs [31,47]. For instance, multiple sexual relationships and other risky sexual behaviours are significantly associated with STIs risk among women and undergraduate university students [38]. A probable explanation for this finding is that this age group often represents a period of increased independence and exploration, both socially and sexually. The quest for self-exploration and identity formation around this period leads many of young adults to experiment sexual behaviours, including casual sex, having multiple partners and inconsistent use of condom [42]. Additionally, individuals in this age range are generally developing greater cognitive maturity and therefore, may still underestimate risks or feel invulnerable to the potential consequences of risky sexual behaviours [42]. Students in level 300 were more likely to suffer STIs relative to those in level 100, whereas those in level 200 and 400 were less likely to suffer STIs compared to others in level 100. This finding is congruent with a study among college students in Motta Town, Northwest Ethiopia which reported 3rd and 2nd year students to be about 5 and 2.5 times more likely to report STIs relative to first year students [39]. Similarly, Gebrekidan et al. [43] found grade 12 students to be about 5 times more likely to acquire STIs compared to those in grade nine in Bahir-Dar high school. Perhaps the reason for level 300s to be more likely to suffer STIs than the level 100s is due the fact that the students in their third year, have developed more independence, confidence, and larger social circles compared to the first-year students, leading to increased sexual activities and experimentation. This can increase their exposure to risky sexual behaviours as documented by CDC [44] and Guttmacher Institute [45] that students who become more comfortable in their university environment are often more likely to engage in such behaviours, increasing their STI risk. Equally, students in levels 200 and 400 might have become more aware of sexual health risks through educational programmes or personal experiences. This increased awareness can lead to more responsible sexual behaviours, such as regular STI testing, use of contraceptives or reduced number of sexual partners [44]. The likelihood of having learned from past mistakes also reduces their susceptibility to contracting STIs [44]. We also believe that in a country and an economy where finding jobs after university graduation is high difficult, level 400 students who are about leaving school may be thinking of how to finding jobs rather than sexual explorations.

We found that non-residential undergraduate students were less vulnerable to STIs compared with those at the traditional halls of residence and university owned auxiliary hostels. This finding is not consistent with earlier findings that students living in rented rooms and apartment were about three times more likely to have multiple sexual partners hence, more likely to suffer STIs [43]. Reasons being that accommodation conditions in the private hostels are more permissive for sexual behaviours relative to the traditional halls of the university where there are rules and regulations guiding the behaviour of students in the traditional halls, but there are little or no rules guiding the behaviours of students in private hostels and accommodations [41]. Thus, students in the private hostels are at much liberty to engage in risky sexual behaviours compared to their counterparts in the university halls of residence. Tura and Alemseged [46] documented that enabling conditions in the surrounding environment where students stay have been associated with risky sexual behaviours and the likelihood of developing STIs.

The observation is that private hostel owners are recently putting in place stringent accommodation conditions that may check behaviours of occupants of their facilities. There are also sexual health campaigns and mobile health interventions have been targeted at non-residential students lately [48]. Moreover, changing dynamics in on-campus living might also be promoting communal living at the traditional halls at the blind side of the school authorities. For example, availability of ingle room accommodations (flats) and greater independence within residence halls provide more opportunities for intimate relationships, contributing to increased STI risk [49]. It is important for university authorities to revise or enforce the existing residence hall policies to encourage responsible behaviours such as visitor restrictions and substance use rules that could contribute to risky behaviours.

Our finding also showed that students who engaged in anal sex were more likely to suffer STIs. This finding is similar to the ones observed in other studies that recorded a significant association between anal sex and an STI positive diagnosis [50]. This may be due the biologically riskier nature of anal sex than other forms of sexual intercourse, because of the structure of the rectum. For example, the rectal tissue are thinner and more prone to tearing which creates entry points for pathogens such as HIV, gonorrhea and syphilis [51]. The delicate nature of the rectal tissues increases the probability of contracting an STI if any of the partner is infected. Unlike vaginal intercourse, the rectum does not naturally produce lubrication, which result in increased friction and a higher chance of tears in the tissue. This makes STI transmission more likely, especially when protective measures such as condoms or lubricants are inconsistently used [51]. Also, the overlapping sexual behaviours of university students may contribute to the higher risk of STIs. That is students who engage in anal sex may also partake in other high risk sexual behaviours such as engaging in sex under the influence of drugs, alcohol or other psychoactive substances that increase their risks. Since such behaviours impair rightful judgment and further reduce the possibility of taking preventive measures before sex [52].

In this current study, undergraduates who do not use condom were more likely to suffer STIs. This finding is in agreement with earlier ones, which revealed that individuals who have never used condoms were 2.4 more likely to acquire STIs than those who used condom [18]. Equally, Muluken [53] documented that, students who have never used condoms during sexual intercourse have higher odds of STIs. Stanger-Hall and Hall [54] investigated sexual risk behaviours in undergraduate populations are noted that non-condom usage remains a significant predictor of STI acquisition, especially among populations engaging in casual sexual encounters. Perhaps, our participants have not been using condoms during every sexual encounter, thus, exposing themselves to infection during those unprotected instances. CDC’s [48] reports on STI trends have highlighted the critical role of condom use in preventing the rise of STIs, mainly in younger populations, which include university students. This call for targeted and regular workshops and seminars that educate students about the importance of condom use and safe sex practices. Also, providing and making available free condoms on campus through deliberate placement of condom dispensers in high traffic areas like hall washrooms, hall infirmaries and lecture theatre washrooms.

Further findings indicated that students who had all their friends to be sexually active were more likely to suffer STIs, while those who had most, some and few of their friends sexually active were less likely to suffer STIs compare to those who do not have sexually active friends. Scholars have reported a very strong association between peer influence, risky sexual behaviours and the occurrence of STIs among young people [55–58]. Perhaps, the principle of homophily may explain this finding, where individuals tend to associate with others who share similar behaviours and attitudes. Therefore, students who have all their friends to be sexually active are more likely to adopt similar behaviours, which could increase their risk of infection with STI [59]. The issue is that friends observe and model sexual behaviours from their peers, which could provide social validation and support for engaging in sexual activity and reinforcing risky behaviours [60]. Hence, there is the need to increase awareness among students through guidance and counselling services about the potential risks of influence from friends who are sexually active.

Moreover, students who have more than one sexual partner were less likely to suffer STIs relative to those who do not currently have sexual partners. This finding does not support previous findings from Ghana [61], Ethiopia [6,62], China [63], South Africa [64] and SSA [65], which documented a positive association between STIs and having multiple sexual partners among men, female sex workers, young people and adolescents. The previous studies found individuals, who had two or more sexual partners to be more likely to report, suffer or diagnosed with STIs. This new finding is surprising because engaging in multiple sexual partnerships is a risky sexual behaviour that increases the odds of contracting STIs [31,63,64,66]. This specific finding are explained by a combination of factors, including safer sexual practices among students who have more partners that are sexually active, differences in testing for STIs and health seeking behaviour of the partners involve in the relationship. The interplay of these factors suggests that individual behaviours in sexual relationships are crucial, rather than the number of partners. As STI testing is becoming common due to youth friendly corners in health facilities in the country and young people are becoming aware of their status, they could therefore be protecting themselves to avoid spreading or contracting infections [67–69].

We also found that students who use drugs always and once in a while during sex were less likely to suffer STIs compare to those who never use drugs during sex. This finding is contrary to previous studies that find drug use to influence engagement in behaviours that increase the chances of individuals’ STI infection [62,70,71]. Andualem et al. [72] reported that people who use drug for sex were about 3 times more likely to be involved in risky sexual behaviours that increase their odds of STIs. Perhaps, the characteristics of the study population explain this variation. We presume that as university students, our participants may be aware of the potential risks, thus, they do take precautions to avoid contracting STIs. Hence, these students may be engaging in risk management practices, such as consistent condom use, even when under the influence of substances. For instance, recreational drug users may adopt harm reduction strategies, including using condoms or engaging in less risky sexual activities [73,74]. Also, this can be as a result of underreporting of sexual behaviours or STI history, residual confounding, reverse causation, or differences in protective sexual practices among some participants. Although these explanations are speculative.

### Limitations

While this study addressed an important public health issue, it has some limitations. First, the cross-sectional design limits our ability to establish causal relationships between the variables. Therefore, the observed associations should be interpreted with caution and should not be regarded as evidence of causality. Additionally, because the survey was conducted within a university setting using convenience (accidental) sampling and voluntary participation, selection bias may have occurred, thereby limiting the generalisability of the findings. Thus, the results may not fully represent the broader population of regular unmarried undergraduate students in Ghana. Another limitation is that STI status was self-reported and was not confirmed through laboratory testing or clinical diagnosis. Given the sensitive nature of sexual health and the cultural and social norms surrounding discussions of sexual behaviour in Ghana, some participants may have underreported or overreported their STI history or sexual behaviours, potentially affecting the validity of the findings. Furthermore, STI status was self-reported by respondents and may therefore be subject to recall and social desirability biases. Given that cultural and social norms in Ghana often make discussions of personal sexual activities sensitive, some respondents may have underreported their sexual behaviours, potentially affecting the validity of our results. Besides, there is a need for future research to include the use of probability-based sampling methods, such as venue-based sampling, to generate more representative estimates of STI prevalence among university students. Moreso, the unexpected finding that students reporting multiple sexual partners and those who used drugs during sexual activity had lower odds of reporting STIs contrasts with the established epidemiological evidence linking these behaviours with an increased risk of STIs. This could be due to underreporting of sexual behaviours or STI history, residual confounding, reverse causation, or differences in protective sexual practices among some participants. However, these explanations are speculative and require further investigation using longitudinal studies and laboratory-confirmed STI data. Finally, the study did not collect data on LGBTQA+ individuals and therefore could not examine STI risk within this population. In addition, the study included only unmarried undergraduate students. Future research would benefit from using a nationwide study with a different study design, incorporating laboratory or clinically confirmed STI diagnoses where possible, and including LGBTQA+ individuals and married students to provide a more comprehensive understanding of STI risk among young people in Ghana.

## Conclusion

The prevalence of risky sexual behaviours among regular unmarried undergraduate university students in Ghana is notably high. STIs among these students is influenced by factors such as age, academic level, sexual preference, non-use of condom, number of sexually active friends, having multiple sexual partners and using drugs for sexual intercourse. These findings underscore the need for health committees, guidance and counseling units and academic faculties to emphasise the risks associated with sexual activities and behaviours to promote healthier behaviours, including healthy sexual practices. Doing so can help prevent STIs, enhance students’ quality of life and well-being, support their academic achievement and to achieve SDG 3 and the global health sector strategy on STIs by 2030. Therefore, effective campus-based counseling, health education and promotion efforts, including peer education are essential to encourage responsible sexual behaviour among the students.

## Supporting information

S1 Data STIs MAnuscript Data. (SAV)
