# Supplementary material for: Prevalence and associated factors of sexually transmitted infections among unmarried undergraduate university students
Source: PLoS One. 2026 Sep 24;21(9):e0358430. doi: 10.1371/journal.pone.0358430 (PMC13607798; doi:10.1371/journal.pone.0358430)
Supplement: Supplementary file 1 [file pone.0358430.s003.pdf]

Dear Editor,

Thank you for your preliminary assessment of our manuscript. Based on the issue raised, we have responded accordingly.

1. Please ensure that you have specified what type you obtained (for instance, written or verbal, and if verbal, how it was documented and witnessed). If your study included minors, state whether you obtained consent from parents or guardians. If the need for consent was waived by the ethics committee, please include this information. The participants are university students whose ages are 18 years and above. They have all signed a written consent form, and that has been indicated in the manuscript on page 3.
