# Supplementary material for: Prevalence and associated factors of sexually transmitted infections among unmarried undergraduate university students
Source: PLoS One. 2026 Sep 24;21(9):e0358430. doi: 10.1371/journal.pone.0358430 (PMC13607798; doi:10.1371/journal.pone.0358430)
Supplement: Supplementary file 2 [file pone.0358430.s004.docx]

**Recommendation**

One of the most commendable features of this study is that it included a huge sample size (10,896) of unmarried undergraduate students, out of a total sample (18,129), making it arguably one of the largest single-institution studies of STI prevalence among university students in Sub-Saharan Africa. The sample has been carefully stratified by academic level (100-400), gender (50:50), and residential status, with balanced representation of key demographic subgroups and strong internal validity of the findings. Alongside this, the study addresses a much-needed void in the literature as it focuses on a population that has been largely underrepresented in Ghana, unmarried university students who are becoming sexually independent, as most of the studies have concentrated on reproductive-age women and adolescents in secondary schools. The study is relevant to public health programming in Ghana and similar low- and middle-income countries, as it addresses both a population and a geographic gap in a developing-country context.

Another advantage is the analytical approach. The authors did not stop at providing descriptive statistics; they also used chi-square tests for two-by-two tables and binary logistic regression with adjusted odds ratios (AORs) and 95% confidence intervals, which is commendable for controlling confounding. Many of the independent variables included cover a wide spectrum of demographic, behavioural, psychosocial, and contextual factors and reflect a holistic understanding of determinants of STIs that align with a social-ecological model of health. The study is well grounded from an ethical standpoint, with IRB approval, written informed consent from all participants, preservation of anonymity, and an innovative, culturally sensitive, practical partitioned canopy setup that ensures privacy in a setting where talking about sexual matters carries social stigma. The data collection is methodologically credible because the questionnaire used is adapted from the Global School-based Student Health Survey (GSHS), which has been pre-tested with more than 300 students from another school. Lastly, the study contextualises the findings within well-established policy frameworks, namely the United Nations Sustainable Development Goal (SDG) 3 and the WHO Global Health Sector Strategy on STIs 2016–2021, highlighting the research's relevance to the global agenda of action and the potential impact it may have on institutional health programming or national STI control action plans.

While this is a considerable strength, the study has several significant weaknesses that need to be addressed. The most important methodological weakness is the complete dependence on self-reported STI diagnosis as the dependent variable, with no laboratory or clinical confirmation by examination. Self-reported STI status can be subject to a number of biases, including recall, social desirability and misclassification bias, especially in a culture where sexual issues are highly charged. Individuals might not want to admit to having an STI out of embarrassment, or they might self-diagnose by relying on symptoms. This will essentially render the estimate of 36.5% prevalence invalid. This should be recognised as the main limitation, and future studies should consider using self-reported data in conjunction with biological STI screening (e.g., chromosomal nucleic acid amplification testing (CHD NAAT) for chlamydia and gonorrhoea, as well as serology for syphilis and HIV) to provide clinically valid prevalence data. A simple yes/no question to differentiate clinician-confirmed infections from self-reported infections would partially address this concern.

Added to this is the fact that accidental convenience sampling was used for the subjects, which, although quite logical for the topic, still introduces substantial selection bias and compromises external validity. The authors modestly admit that students were more inclined to volunteer if they were concerned about STIs. This was a self-selection and almost certainly overestimated the prevalence rate. It is important to caution readers when comparing findings with those of studies using probability-based random sampling, such as the Gondar University study, which reported a 18.2% prevalence. Cluster or venue sampling methods, rather than relying on voluntary self-selection, could be used in future studies to provide more representative and generalizable estimates. The study does not sufficiently account for the shift from a total of 18,129 recruited students to 10,896 analysed, nor does it present refusal rates or the number of incomplete questionnaires, nor does it explain how missing data is handled. To evaluate the possibility of systematic non-response bias, a STROBE-compliant participant flow diagram and reporting of data completeness must be present.

The cross-sectional design is recognised as a limitation of the study but receives little attention beyond the brief mention in the limitations section. Due to this design, it is not possible to establish temporal or causal relationships between identified risk factors and the acquisition of STIs, although this has direct implications for the article's design. This is especially concerning because of two counterintuitive results: first, students who had multiple sex partners and those who use drugs at sex seemed less likely to develop STIs, results that are in opposition to decades of global epidemiological data. What the authors speculate, that users might take harm reduction measures or that students with more partners may engage in safer sex, are logical possibilities, but there is no data in this study that supports either possibility. These results could also reflect a methodological effect related to self-reported outcome bias, as they may be due to reverse causation or to residual confounding from uncontrolled factors (e.g., relationship type). The authors should give these findings greater analytical focus; stratified analyses or interaction testing should be conducted, and it should be clearly stated that these findings should not be used to guide public health messaging until further longitudinal research is conducted.

The manuscript also contains inconsistencies and errors in the presentation of results, which detract from its credibility. The 36.5% prevalence figure is based on 10,896 students in the abstract, whereas the chi-square analysis in Table 3 is based on a sub-sample of 4,124 students (with no explanation for the discrepancy). It is presumed that this is due to the fact that these students were sexually active, but the article does not report a number of sexually active and inactive students, nor a number of sexually active students in the prevalence denominator and regression analyses. The denominator should exclude sexually inactive individuals because their prevalence would be artificially lowered, and the denominator should exclude them from risk factor analyses because this would artificially inflate the odds ratios. There is a need for a separate, well-reported prevalence estimate among only sexually active students. In addition, a number of apparent misalignments in the labelling of the B values, AORs and academic level categories are present in Table 4; the 95% CI for the finding at academic level 300 (.785–1.373) crosses 1.0, suggesting that the finding is not statistically significant, but is reported as such in the abstract and in the body of the text. All these errors must be carefully corrected by a careful cross-check of all tables with the narrative.

Statistically, the logistic regression model has an important issue that is not addressed here: the Hosmer-Lemeshow goodness-of-fit test yielded a p-value of 0.004, indicating a poor fit (p-value > 0.05 is the desired value). The authors do not speak to this fact, which is a significant analytical deficiency. Also, the model does not include any checks for multicollinearity, although there are some overlapping constructs, including the composite variable "sexual behaviour" and the separate constructs "condom use," "sexual preference", and "drug use," which are likely correlated. The unexpected findings may be due to multicollinearity, which can elevate standard errors and make AOR results unstable. The authors should either re-specify their model to satisfy the goodness-of-fit criterion, or report the variance inflation factors or discuss the consequences of failing to meet the goodness-of-fit test for the validity of the regression results.

Theoretically, the study lacks a well-developed theoretical orientation for selecting variables, and there is no clear guidance for understanding the findings. The wide range of independent variables investigated seems somewhat ad hoc, and the lack of a common conceptual framework (such as the Health Belief Model, Theory of Planned Behaviour, or Social-Ecological Model) undermines the report's conceptual unity. The results would be more theoretically relevant and could be presented as mechanisms if the study were based on an existing framework. It is particularly noticeable when discussing peer influence and parent communication, which are analysed without reference to social norm theory or social learning theory; both, if applied, would significantly enrich the interpretation. Last, rereading the discussion on religious affiliation as a risk factor would benefit from more nuance and more careful citation. The results that Muslim students were more likely to have STIs than Christians is an important finding and should be approached sensitively and with evidence from peer-reviewed literature regarding religion and sexual behaviour and STIs in the African context. The lay health source used to support this is not suitable for a PLoS One submission and needs to be replaced with strong peer-reviewed sources (Yen, 2021, on birth control and religion).

Overall, this study is a valuable, timely piece of research that contributes to the body of knowledge on STI burden among university students in Ghana, particularly through its breadth, the range of variables included, and its policy recommendations. But there is a lot more that needs to be rewritten before it's ready for publication. The authors should pay special attention to correcting inconsistencies in the results table, clarifying the logistic regression model, providing a more complete discussion of the counterintuitive results, explaining the analytical sub-sample for the logistic regression, and further developing the study's theory. Addressing these weaknesses would significantly increase the scientific merit and value of the article.
