# Supplementary material for: Prevalence and associated factors of sexually transmitted infections among unmarried undergraduate university students
Source: PLoS One. 2026 Sep 24;21(9):e0358430. doi: 10.1371/journal.pone.0358430 (PMC13607798; doi:10.1371/journal.pone.0358430)
Supplement: Supplementary file 3 [file pone.0358430.s005.docx]

**RESPONSES TO REVIEWERS’ COMMENTS**

The authors thank the reviewers for their remarks and expert reviews in making our work clearer and improving its quality.

We provide a point-by-point response to the comments and suggestions of the reviewers.

| **Reviewer’s** **comments** | **Responses** |
| --- | --- |
| **Reviewer** **1** |  |
| Comment 1  Consider editing the results section of the abstract to improve its clarity and alignment with the objectives of the study. It may not be clear to all readers what levels 200 and Levels 300 mean in the results section. | Thank you for the concise observation on the abstract. However, we have carefully made revisions in the abstract as identified. The levels 100, 200, 300 and 400 used to categorised undergraduates in this study is analogous to freshman, sophomore, junior and senior in the United States. While Ghanaian universities use levels 100, 200, 300 and 400 or 1st, 2nd, 3rd, and 4th years for undergraduate categorisation, U.S. uses freshman, sophomore, junior and senior. This revision can be found on Page 1, lines 30-32. |
| Comment 2  The fourth sentence under background is strongly judgmental, stating as follows: STIs are accountable for the burden of morbidity and mortality in many countries globally, particularly among young people, because of their unhealthy lifestyles. Consider editing it to read as follows:  ..STIs contribute substantially to morbidity and mortality globally, particularly among young people, due to a range of behavioral and structural risk factors. | Thank you for this important observation. We have revised the sentence, and it can be found on Page 2, lines 56 and 57. |
| Comment 3  The methods section needs major revision. It is unclear how the final analytic sample was reduced from 18,129 to 10,896 participants. The authors should clearly describe the sampling process, inclusion and exclusion criteria, and any data cleaning or missing data procedures that led to this reduction.  In addition, the methods lack enough detail to allow replication. Please provide clear information on study design, participant selection, data collection procedures, variable definitions, and statistical analyses. As currently written, another researcher would not be able to reproduce the study independently. | We sincerely thank you for this constructive comment. The 18,129 is the total undergraduate student population of the school, out of which 10, 896 was selected, representing approximately 60% sample size of the study.  We have therefore extensively revised the Methods section to enhance its clarity, transparency, and reproducibility. Specifically, we have provided a clear explanation of how the final analytic sample of 10,896 participants was derived from the total population of 18,129 undergraduate students of the institution used for the study by describing the inclusion and exclusion criteria and the participant selection process. We have also expanded the description of the study design, data source, participant selection, and data collection procedures and provided clearer operational definitions and coding of the outcome and explanatory variables. In addition, the Statistical Analysis subsection is amended to provide a detailed description of analytical procedures, including descriptive, bivariate, and multivariable analyses performed, the criteria used for variable selection, measures of association reported, the statistical significance level, and the statistical software used. These revisions can be found on pages 3 and 4, lines 137 and 145 – 192. |
| Comment 4  STI status was based on self-report, but it is not clear how a common understanding of “STI” was ensured among participants. Please clarify whether a standard definition, examples, or any guidance was provided during data collection to improve consistency and reduce misclassification.  In addition, it would be helpful to explain how self-reported responses were categorized into specific conditions such as gonorrhea and syphilis. For example, did participants report a prior clinical diagnosis, symptoms, or were they given a list of infections to select from? Providing these details would strengthen confidence in the validity of the outcome measure. | We acknowledge the reviewer for this insightful comment. In response, we have revised the Measures section to provide additional detail on the assessment of sexually transmitted infections (STIs). We clarified how STI status was defined and measured during data collection, including the information and guidance provided to participants to promote a common understanding of the term "STI" to minimise potential misclassification. We have also expanded the description of the outcome measure to explain how self-reported responses were classified into specific STI conditions, including whether participants reported a previous clinical diagnosis and/or selected from a predefined list of STI types. These revisions improve the transparency of the outcome measurement and strengthen readers' confidence in the validity and interpretation of the self-reported STI data. This revision can be found on page 5, lines 207-232. |
| Comment 5  Discussion  In the first paragraph under discussion, the following sentence comes across as judgmental and speculative. You can make the same point in a neutral way by focusing on behaviors and context rather than labeling students. You may consider re-writing it to read something like this:  These findings may point to increased vulnerability to risky or potentially exploitative sexual situations among students in this group. | We appreciate you for this thoughtful suggestion. We agree that the original wording could be perceived as judgmental and speculative. To respond to that, we reworked the first paragraph of the Discussion using more neutral language. We replaced the original statement with wording that indicates that the findings "may point to increased vulnerability to risky or potentially exploitative sexual situations among students in this group." This revision can be found on Page 10, lines 321-314. |
| **Reviewer** **2** |  |
| Comment 1  …The most important methodological weakness is the complete dependence on self-reported STI diagnosis as the dependent variable, with no laboratory or clinical confirmation by examination. Self-reported STI status can be subject to a number of biases, including recall, social desirability and misclassification bias, especially in a culture where sexual issues are highly charged. Individuals might not want to admit to having an STI out of embarrassment, or they might self-diagnose by relying on symptoms. This will essentially render the estimate of 36.5% prevalence invalid. This should be recognised as the main limitation, and future studies should consider using self-reported data in conjunction with biological STI screening… | We thank you for this important observation. We agree that the study has a methodological limitation because the STI status was based on self-reported information without laboratory or clinical confirmation. To address this, the Limitations sections have been significantly updated to clearly recognise that self-reported STI diagnosis may be prone to recall bias, social desirability bias and possible misclassification issues, especially in the context of sensitive sexual health topics. We have also tempered the interpretation of the reported STI prevalence by emphasising that the validity may be affected due to these inherent limitations. Moreover, recommend that future studies should supplement self-reported STI data with laboratory or clinically confirmed STI screening to enhance the validity and reliability of STI prevalence estimates and analyses of STI-related risk factors. This revision can be found on be Page 13, lines 461-481 |
| Comment 2  Added to this is the fact that accidental convenience sampling was used for the subjects, which, although quite logical for the topic, still introduces substantial selection bias and compromises external validity. The authors modestly admit that students were more inclined to volunteer if they were concerned about STIs. This was a self-selection and almost certainly overestimated the prevalence rate. It is important to caution readers when comparing findings with those of studies using probability-based random sampling, such as the Gondar University study, which reported a 18.2% prevalence. Cluster or venue sampling methods, rather than relying on voluntary self-selection, could be used in future studies to provide more representative and generalizable estimates. | Thank you for highlighting this important methodological consideration. We agree that the use of convenience sampling is a limitation of the study, as voluntary participation may introduce selection bias and limit the external validity and generalisability of the findings. In response, we have revised the Limitations section to provide a more explicit discussion of the potential influence of self-selection bias, acknowledging that students who perceived themselves to be at greater risk of STIs or had greater interest in sexual health issues may have been more likely to participate, which could have influenced the observed prevalence estimate. Additionally, we have highlighted the need for future research to include the use of probability-based sampling methods, such as venue-based sampling, to generate more representative estimates of STI prevalence among university students. This revision can be found in the limitations section of the manuscript (Page 13, lines 462-482) |
| Comment 3  The study does not sufficiently account for the shift from a total of 18,129 recruited students to 10,896 analysed, nor does it present refusal rates or the number of incomplete questionnaires, nor does it explain how missing data is handled. To evaluate the possibility of systematic non-response bias, a STROBE-compliant participant flow diagram and reporting of data completeness must be present. | Thank you for this important observation. We agree that the final analytic sample was derived in a manner that needed to be clarified. To address this, we have updated the Methods section to explicitly detail the process of sample selection and why the 10,896 sample size was selected from the total unmarried undergraduate student population of 18,129. These revisions can be found on pages 3 and 4, lines 137 and 145 – 192. |
| **Comment** 4  The cross-sectional design is recognised as a limitation of the study but receives little attention beyond the brief mention in the limitations section. Due to this design, it is not possible to establish temporal or causal relationships between identified risk factors and the acquisition of STIs, although this has direct implications for the article's design. This is especially concerning because of two counterintuitive results: first, students who had multiple sex partners and those who use drugs at sex seemed less likely to develop STIs, results that are in opposition to decades of global epidemiological data. What the authors speculate, that users might take harm reduction measures or that students with more partners may engage in safer sex, are logical possibilities, but there is no data in this study that supports either possibility. These results could also reflect a methodological effect related to self-reported outcome bias, as they may be due to reverse causation or to residual confounding from uncontrolled factors (e.g., relationship type). The authors should give these findings greater analytical focus; stratified analyses or interaction testing should be conducted, and it should be clearly stated that these findings should not be used to guide public health messaging until further longitudinal research is conducted. | Thank you for this insightful and important comment. It is acknowledged that the cross-sectional design has key limitations in interpreting the observed association, especially in terms of temporal relationships and causal associations between identified factors and STI status. We have also updated the Discussion and Limitations sections to offer a fuller explanation of the implications of the study design and to stress that the associations that have been found must be understood as correlational and not causal. We have also revised the interpretation of the unexpected findings regarding multiple sexual partners and drug use during sexual activity to ensure a more cautious and balanced discussion. We recognise that such results may be due to potential reverse causation, residual confounding, reporting bias, and/or other factors not measured, and that the explanations offered in our prior version were hypotheses, not conclusions based on the available data. Moreover, we have argued that the conclusions that these studies draw must not become the basis for altering public health guidelines until they can be backed by longitudinal studies and more intensive epidemiological investigations. We appreciate the reviewer’s suggestion regarding additional analyses and have considered this recommendation; however, because of limitations in the available dataset and the exploratory nature of these associations, further stratified or interaction analyses were not conducted. Instead, we have strengthened the discussion to appropriately contextualise these results and highlight the need for future studies using longitudinal designs and more comprehensive measurement approaches. These revisions can be found on Page 13, lines 463-489. |
| **Comment 5**  The manuscript also contains inconsistencies and errors in the presentation of results, which detract from its credibility. The 36.5% prevalence figure is based on 10,896 students in the abstract, whereas the chi-square analysis in Table 3 is based on a sub-sample of 4,124 students (with no explanation for the discrepancy). It is presumed that this is due to the fact that these students were sexually active, but the article does not report a number of sexually active and inactive students, nor a number of sexually active students in the prevalence denominator and regression analyses. The denominator should exclude sexually inactive individuals because their prevalence would be artificially lowered, and the denominator should exclude them from risk factor analyses because this would artificially inflate the odds ratios. | We thank you for this important observation. We agree that the distinction between the overall survey sample and the analytical sample was not sufficiently clear in the previous version of the manuscript. In the revised manuscript, we have clarified that although the study included 10,896 unmarried regular undergraduate students in the initial dataset, analyses related to STIs, including prevalence estimation and associated factors, were restricted to students who reported being sexually active (n = 4,124), as only these individuals were considered at risk of experiencing STI-related outcomes. We now explicitly report the number of sexually active and sexually inactive students in the Results section; we also clarified the denominator used for the prevalence estimate and multivariable logistic regression analyses. This can be found on page 1 lines 27 – 30. |
| **Comment** 6  There is a need for a separate, well-reported prevalence estimate among only sexually active students. In addition, a number of apparent misalignments in the labelling of the B values, AORs and academic level categories are present in Table 4; the 95% CI for the finding at academic level 300 (.785–1.373) crosses 1.0, suggesting that the finding is not statistically significant, but is reported as such in the abstract and in the body of the text. All these errors must be carefully corrected by a careful cross-check of all tables with the narrative. | Thank you for this careful observation. To verify the accuracy of all reported regression coefficients, adjusted odds ratios, 95% confidence intervals, and p-values, we cross-checked the original statistical output against Table 4 and the accompanying narrative. During this review, we confirmed that the 95% confidence interval of 0.785–1.373 corresponds to academic Level 200, which is correctly reported as not statistically significant (p = 0.793), whereas academic Level 300 has a 95% confidence interval of 0.371–0.565 (p < 0.001), indicating a statistically significant association. To avoid any possible confusion, we reviewed the formatting and labelling of Table 4 and ensured that both the table and the accompanying narrative are fully aligned and accurately reflect the original statistical output. This revision can be found on Page 8, Table 4. |
| **Comment 7**  Statistically, the logistic regression model has an important issue that is not addressed here: the Hosmer-Lemeshow goodness-of-fit test yielded a p-value of 0.004, indicating a poor fit (p-value > 0.05 is the desired value). The authors do not speak to this fact, which is a significant analytical deficiency. Also, the model does not include any checks for multicollinearity, although Reviewers' comments- Marythere are some overlapping constructs, including the composite variable "sexual behaviour" and the separate constructs "condom use," "sexual preference", and "drug use," which are likely correlated. The unexpected findings may be due to multicollinearity, which can elevate standard errors and make AOR results unstable. The authors should either re-specify their model to satisfy the goodness-of-fit criterion, or report the variance inflation factors or discuss the consequences of failing to meet the goodness-of-fit test for the validity of the regression results. | We appreciate your valuable comment regarding the logistic regression model. In response, we reassessed the model fit and evaluated multicollinearity among the predictor variables using variance inflation factors (VIFs), which did not indicate problematic multicollinearity. We also acknowledge that the Hosmer-Lemeshow goodness-of-fit test indicated that the model did not fit the data optimally. We have therefore modified the manuscript to explicitly report this finding and have restricted the regression analysis with this as a limitation. We have also provided a sensible interpretation of the adjusted odds ratios and tempered the conclusions from the multivariable model accordingly. This can be found on Page 8 and 9, lines 287, 288 and 293. |
| **Comment** 8  this is another comment: Theoretically, the study lacks a well-developed theoretical orientation for selecting variables, and there is no clear guidance for understanding the findings. The wide range of independent variables investigated seems somewhat ad hoc, and the lack of a common conceptual framework (such as the Health Belief Model, Theory of Planned Behaviour, or Social-Ecological Model) undermines the report's conceptual unity. The results would be more theoretically relevant and could be presented as mechanisms if the study were based on an existing framework. It is particularly noticeable when discussing peer influence and parent communication, which are analysed without reference to social norm theory or social learning theory; both, if applied, would significantly enrich the interpretation | Thank you very much for this valuable observation. We agree that the previous version failed to clearly explain the rationale behind the choice and interpretation of the variables in the study with the help of a theory. In response, we have included a theoretical framework by incorporating the Social Ecological Model (SEM). The revised manuscript now explains how individual-level factors, interpersonal-level factors and behavioural factors may interact to influence STI risk. This revision can be found on page 3, lines 107-133 |
| **Reviewer 3** |  |
| **Comment 1**  The authors should justify the use of convenience sampling and explain the potential impact on the overall sample. | Thank you for this important observation. We have thoroughly made this correction, and this revision can be found on Page 4, lines 160-163 |
| Describe how the authors validated the questionnaire and the process behind the "sexual behavior" construct | The questionnaire used in this study was adapted from previously validated instruments assessing sexual and reproductive health behaviours among young people. Before data collection, the questionnaire was reviewed by experts in public health, epidemiology, and sexual and reproductive health to assess its content relevance, clarity, and appropriateness. The revised instrument was subsequently pre-tested among a sample of students with characteristics similar to those of the study population to evaluate item clarity, comprehension, and administration procedures. Feedback obtained during the pre-test informed minor revisions to improve the wording and sequencing of the questions before the final survey was administered. The revision can be found on page 5, lines 201–205). |
| Define how STIs are reported (self-report vs. clinical diagnosis) and explain potential misclassification and social desirability bias. Address the unexpected result of lower STIs associated with multiple partners and drug use by presenting other potential explanations and describe them as speculative. | Thank you very much for highlighting this important methodological consideration. We agree that the use of convenience sampling is a limitation of the study, as voluntary participation may introduce selection bias and limit the external validity and generalisability of the findings. In response, we have revised the Limitations section to provide a more explicit discussion of the potential influence of self-selection bias. Additionally, we have highlighted the need for future research to include the use of probability-based sampling methods, venue-based sampling, to generate more representative estimates of STI prevalence among university students. We have also established that although convenience sampling may have an impact on representativeness, its direction and magnitude on prevalence estimates cannot be determined from the current study alone. This revision can be found in the limitations section of the manuscript (Page 13, lines 463-489) |
