# Supplementary material for: Prevalence and associated factors of sexually transmitted infections among unmarried undergraduate university students
Source: PLoS One. 2026 Sep 24;21(9):e0358430. doi: 10.1371/journal.pone.0358430 (PMC13607798; doi:10.1371/journal.pone.0358430)
Supplement: Peer review history

# Author comment: Author response to Decision Letter 0

**DOI:** 10.1371/journal.pone.0358430.r001

17 Dec 2025

Attachment Submitted filename: Response to Editor comments.pdf

# Peer review: Decision Letter 0

**DOI:** 10.1371/journal.pone.0358430.r002

**Authors:** Harikrishnan U

31 May 2026

Dear Dr. Hormenu,

Thank you for submitting your manuscript to PLOS ONE. After careful consideration, we feel that it has merit but does not fully meet PLOS ONE’s publication criteria as it currently stands. Therefore, we invite you to submit a revised version of the manuscript that addresses the points raised during the review process.

Please submit your revised manuscript by Jul 15 2026 11:59PM. If you will need more time than this to complete your revisions, please reply to this message or contact the journal office at plosone@plos.org. When you're ready to submit your revision, log on to https://www.editorialmanager.com/pone/ and select the 'Submissions Needing Revision' folder to locate your manuscript file.

- A letter that responds to each point raised by the academic editor and reviewer(s). You should upload this letter as a separate file labeled 'Response to Reviewers'.
- A marked-up copy of your manuscript that highlights changes made to the original version. You should upload this as a separate file labeled 'Revised Manuscript with Track Changes'.
- An unmarked version of your revised paper without tracked changes. You should upload this as a separate file labeled 'Manuscript'.

If you would like to make changes to your financial disclosure, please include your updated statement in your cover letter. Guidelines for resubmitting your figure files are available below the reviewer comments at the end of this letter.

If applicable, we recommend that you deposit your laboratory protocols in protocols.io to enhance the reproducibility of your results. Protocols.io assigns your protocol its own identifier (DOI) so that it can be cited independently in the future. For instructions see: https://journals.plos.org/plosone/s/submission-guidelines#loc-laboratory-protocols. Additionally, PLOS ONE offers an option for publishing peer-reviewed Lab Protocol articles, which describe protocols hosted on protocols.io. Read more information on sharing protocols at https://plos.org/protocols?utm_medium=editorial-email&utm_source=authorletters&utm_campaign=protocols.

As the corresponding author, your ORCID iD is verified in the submission system and will appear in the published article. PLOS supports the use of ORCID, and we encourage all coauthors to register for an ORCID iD and use it as well. Please encourage your coauthors to verify their ORCID iD within the submission system before final acceptance, as unverified ORCID iDs will not appear in the published article. *Only*  the individual author can complete the verification step; PLOS staff *cannot*  verify ORCID iDs on behalf of authors.

We look forward to receiving your revised manuscript.

Kind regards,

Harikrishnan U, Ph.D

Academic Editor

PLOS One

Journal Requirements:

When submitting your revision, we need you to address these additional requirements.

1. Please ensure that your manuscript meets PLOS ONE's style requirements, including those for file naming. The PLOS ONE style templates can be found at

https://journals.plos.org/plosone/s/file?id=wjVg/PLOSOne_formatting_sample_main_body.pdf and

https://journals.plos.org/plosone/s/file?id=ba62/PLOSOne_formatting_sample_title_authors_affiliations.pdf

2. In the online submission form you indicate that your data is not available for proprietary reasons and have provided a contact point for accessing this data. Please note that your current contact point is a co-author on this manuscript. According to our Data Policy, the contact point must not be an author on the manuscript and must be an institutional contact, ideally not an individual. Please revise your data statement to a non-author institutional point of contact, such as a data access or ethics committee, and send this to us via return email. Please also include contact information for the third party organization, and please include the full citation of where the data can be found.

3. Please amend the manuscript submission data (via Edit Submission) to include author Jacob Owusu Sarfo.

4. Your ethics statement should only appear in the Methods section of your manuscript. If your ethics statement is written in any section besides the Methods, please delete it from any other section.

5. If the reviewer comments include a recommendation to cite specific previously published works, please review and evaluate these publications to determine whether they are relevant and should be cited. There is no requirement to cite these works unless the editor has indicated otherwise.

[Note: HTML markup is below. Please do not edit.]

Reviewers' comments:

Reviewer's Responses to Questions

**Comments to the Author**

1. Is the manuscript technically sound, and do the data support the conclusions?

Reviewer #1: Yes

Reviewer #2: Yes

Reviewer #3: Partly

**********

2. Has the statistical analysis been performed appropriately and rigorously?

Reviewer #1: Yes

Reviewer #2: Yes

Reviewer #3: I Don't Know

**********

3. Have the authors made all data underlying the findings in their manuscript fully available?

The PLOS Data policy

Reviewer #1: Yes

Reviewer #2: Yes

Reviewer #3: Yes

**********

4. Is the manuscript presented in an intelligible fashion and written in standard English?

Reviewer #1: Yes

Reviewer #2: Yes

Reviewer #3: Yes

**********

Reviewer #1: Summary

The authors investigated the prevalence and factors associated with sexually transmitted infections among young, unmarried undergraduate university students in Ghana. They collected data on sexual behaviors that contribute to HIV/STI and unintended pregnancies from 10,896 participants. About 37% reported having had at least one STI, with gonorrhea and syphilis being the most common. The authors recommended institution-based health education and promotion programs to lower the risk of STIs, including HIV/AIDS among the students.

Overall Comment

The manuscript is generally well written and provides useful information on prevalence and factors associated with STI among university students in Ghana. However, it may benefit from some edits to strengthen it and improve clarity.

Abstract:

Consider editing the results section of the abstract to improve its clarity and alignment with the objectives of the study. It may not be clear to all readers what levels 200 and Levels 300 mean in the results section.

Background

The fourth sentence under background is strongly judgmental, stating as follows .. STIs are accountable for the burden of morbidity and mortality in many countries globally, particularly among young people, because of their unhealthy lifestyles. Consider editing it to read as follows:

‥STIs contribute substantially to morbidity and mortality globally, particularly among young people, due to a range of behavioral and structural risk factors.

Methods

The methods section needs major revision. It is unclear how the final analytic sample was reduced from 18,129 to 10,896 participants. The authors should clearly describe the sampling process, inclusion and exclusion criteria, and any data cleaning or missing data procedures that led to this reduction.

In addition, the methods lack enough detail to allow replication. Please provide clear information on study design, participant selection, data collection procedures, variable definitions, and statistical analyses. As currently written, another researcher would not be able to reproduce the study independently.

Measures

STI status was based on self-report, but it is not clear how a common understanding of “STI” was ensured among participants. Please clarify whether a standard definition, examples, or any guidance was provided during data collection to improve consistency and reduce misclassification.

In addition, it would be helpful to explain how self-reported responses were categorized into specific conditions such as gonorrhea and syphilis. For example, did participants report a prior clinical diagnosis, symptoms, or were they given a list of infections to select from? Providing these details would strengthen confidence in the validity of the outcome measure.

Discussion

In the first paragraph under discussion, the following sentence comes across as judgmental and speculative. You can make the same point in a neutral way by focusing on behaviors and context rather than labeling students. You may consider re-writing it to read something like this:

These findings may point to increased vulnerability to risky or potentially exploitative sexual situations among students in this group.

Reviewer #2: Recommendation

One of the most commendable features of this study is that it included a huge sample size (10,896) of unmarried undergraduate students, out of a total sample (18,129), making it arguably one of the largest single-institution studies of STI prevalence among university students in Sub-Saharan Africa. The sample has been carefully stratified by academic level (100-400), gender (50:50), and residential status, with balanced representation of key demographic subgroups and strong internal validity of the findings. Alongside this, the study addresses a much-needed void in the literature as it focuses on a population that has been largely underrepresented in Ghana, unmarried university students who are becoming sexually independent, as most of the studies have concentrated on reproductive-age women and adolescents in secondary schools. The study is relevant to public health programming in Ghana and similar low- and middle-income countries, as it addresses both a population and a geographic gap in a developing-country context.

Another advantage is the analytical approach. The authors did not stop at providing descriptive statistics; they also used chi-square tests for two-by-two tables and binary logistic regression with adjusted odds ratios (AORs) and 95% confidence intervals, which is commendable for controlling confounding. Many of the independent variables included cover a wide spectrum of demographic, behavioural, psychosocial, and contextual factors and reflect a holistic understanding of determinants of STIs that align with a social-ecological model of health. The study is well grounded from an ethical standpoint, with IRB approval, written informed consent from all participants, preservation of anonymity, and an innovative, culturally sensitive, practical partitioned canopy setup that ensures privacy in a setting where talking about sexual matters carries social stigma. The data collection is methodologically credible because the questionnaire used is adapted from the Global School-based Student Health Survey (GSHS), which has been pre-tested with more than 300 students from another school. Lastly, the study contextualises the findings within well-established policy frameworks, namely the United Nations Sustainable Development Goal (SDG) 3 and the WHO Global Health Sector Strategy on STIs 2016–2021, highlighting the research's relevance to the global agenda of action and the potential impact it may have on institutional health programming or national STI control action plans.

While this is a considerable strength, the study has several significant weaknesses that need to be addressed. The most important methodological weakness is the complete dependence on self-reported STI diagnosis as the dependent variable, with no laboratory or clinical confirmation by examination. Self-reported STI status can be subject to a number of biases, including recall, social desirability and misclassification bias, especially in a culture where sexual issues are highly charged. Individuals might not want to admit to having an STI out of embarrassment, or they might self-diagnose by relying on symptoms. This will essentially render the estimate of 36.5% prevalence invalid. This should be recognised as the main limitation, and future studies should consider using self-reported data in conjunction with biological STI screening (e.g., chromosomal nucleic acid amplification testing (CHD NAAT) for chlamydia and gonorrhoea, as well as serology for syphilis and HIV) to provide clinically valid prevalence data. A simple yes/no question to differentiate clinician-confirmed infections from self-reported infections would partially address this concern.

Added to this is the fact that accidental convenience sampling was used for the subjects, which, although quite logical for the topic, still introduces substantial selection bias and compromises external validity. The authors modestly admit that students were more inclined to volunteer if they were concerned about STIs. This was a self-selection and almost certainly overestimated the prevalence rate. It is important to caution readers when comparing findings with those of studies using probability-based random sampling, such as the Gondar University study, which reported a 18.2% prevalence. Cluster or venue sampling methods, rather than relying on voluntary self-selection, could be used in future studies to provide more representative and generalizable estimates. The study does not sufficiently account for the shift from a total of 18,129 recruited students to 10,896 analysed, nor does it present refusal rates or the number of incomplete questionnaires, nor does it explain how missing data is handled. To evaluate the possibility of systematic non-response bias, a STROBE-compliant participant flow diagram and reporting of data completeness must be present.

The cross-sectional design is recognised as a limitation of the study but receives little attention beyond the brief mention in the limitations section. Due to this design, it is not possible to establish temporal or causal relationships between identified risk factors and the acquisition of STIs, although this has direct implications for the article's design. This is especially concerning because of two counterintuitive results: first, students who had multiple sex partners and those who use drugs at sex seemed less likely to develop STIs, results that are in opposition to decades of global epidemiological data. What the authors speculate, that users might take harm reduction measures or that students with more partners may engage in safer sex, are logical possibilities, but there is no data in this study that supports either possibility. These results could also reflect a methodological effect related to self-reported outcome bias, as they may be due to reverse causation or to residual confounding from uncontrolled factors (e.g., relationship type). The authors should give these findings greater analytical focus; stratified analyses or interaction testing should be conducted, and it should be clearly stated that these findings should not be used to guide public health messaging until further longitudinal research is conducted.

The manuscript also contains inconsistencies and errors in the presentation of results, which detract from its credibility. The 36.5% prevalence figure is based on 10,896 students in the abstract, whereas the chi-square analysis in Table 3 is based on a sub-sample of 4,124 students (with no explanation for the discrepancy). It is presumed that this is due to the fact that these students were sexually active, but the article does not report a number of sexually active and inactive students, nor a number of sexually active students in the prevalence denominator and regression analyses. The denominator should exclude sexually inactive individuals because their prevalence would be artificially lowered, and the denominator should exclude them from risk factor analyses because this would artificially inflate the odds ratios. There is a need for a separate, well-reported prevalence estimate among only sexually active students. In addition, a number of apparent misalignments in the labelling of the B values, AORs and academic level categories are present in Table 4; the 95% CI for the finding at academic level 300 (.785–1.373) crosses 1.0, suggesting that the finding is not statistically significant, but is reported as such in the abstract and in the body of the text. All these errors must be carefully corrected by a careful cross-check of all tables with the narrative.

Statistically, the logistic regression model has an important issue that is not addressed here: the Hosmer-Lemeshow goodness-of-fit test yielded a p-value of 0.004, indicating a poor fit (p-value > 0.05 is the desired value). The authors do not speak to this fact, which is a significant analytical deficiency. Also, the model does not include any checks for multicollinearity, although there are some overlapping constructs, including the composite variable "sexual behaviour" and the separate constructs "condom use," "sexual preference", and "drug use," which are likely correlated. The unexpected findings may be due to multicollinearity, which can elevate standard errors and make AOR results unstable. The authors should either re-specify their model to satisfy the goodness-of-fit criterion, or report the variance inflation factors or discuss the consequences of failing to meet the goodness-of-fit test for the validity of the regression results.

Theoretically, the study lacks a well-developed theoretical orientation for selecting variables, and there is no clear guidance for understanding the findings. The wide range of independent variables investigated seems somewhat ad hoc, and the lack of a common conceptual framework (such as the Health Belief Model, Theory of Planned Behaviour, or Social-Ecological Model) undermines the report's conceptual unity. The results would be more theoretically relevant and could be presented as mechanisms if the study were based on an existing framework. It is particularly noticeable when discussing peer influence and parent communication, which are analysed without reference to social norm theory or social learning theory; both, if applied, would significantly enrich the interpretation. Last, rereading the discussion on religious affiliation as a risk factor would benefit from more nuance and more careful citation. The results that Muslim students were more likely to have STIs than Christians is an important finding and should be approached sensitively and with evidence from peer-reviewed literature regarding religion and sexual behaviour and STIs in the African context. The lay health source used to support this is not suitable for a PLoS One submission and needs to be replaced with strong peer-reviewed sources (Yen, 2021, on birth control and religion).

Overall, this study is a valuable, timely piece of research that contributes to the body of knowledge on STI burden among university students in Ghana, particularly through its breadth, the range of variables included, and its policy recommendations. But there is a lot more that needs to be rewritten before it's ready for publication. The authors should pay special attention to correcting inconsistencies in the results table, clarifying the logistic regression model, providing a more complete discussion of the counterintuitive results, explaining the analytical sub-sample for the logistic regression, and further developing the study's theory. Addressing these weaknesses would significantly increase the scientific merit and value of the article.

Reviewer #3: The authors should justify the use of convenience sampling and explain the potential impact on the overall sample. Describe how the authors validated the questionnaire and the process behind the "sexual behavior" construct.

Define how STIs are reported (self-report vs. clinical diagnosis) and explain potential misclassification and social desirability bias. Address the unexpected result of lower STIs associated with multiple partners and drug use by presenting other potential explanations and describe them as speculative.

**********

what does this mean?). If published, this will include your full peer review and any attached files.

If you choose “no”, your identity will remain anonymous but your review may still be made public.

**Do you want your identity to be public for this peer review?**  For information about this choice, including consent withdrawal, please see our Privacy Policy

Reviewer #1: No

Reviewer #2: No

Reviewer #3: No

**********

[NOTE: If reviewer comments were submitted as an attachment file, they will be attached to this email and accessible via the submission site. Please log into your account, locate the manuscript record, and check for the action link "View Attachments". If this link does not appear, there are no attachment files.]

To ensure your figures meet our technical requirements, please review our figure guidelines: https://journals.plos.org/plosone/s/figures

You may also use PLOS’s free figure tool, NAAS, to help you prepare publication quality figures: https://journals.plos.org/plosone/s/figures#loc-tools-for-figure-preparation.

NAAS will assess whether your figures meet our technical requirements by comparing each figure against our figure specifications.

Attachment Submitted filename: Recommendation.docx

# Author comment: Author response to Decision Letter 1

**DOI:** 10.1371/journal.pone.0358430.r003

10 Aug 2026

We thank reviewers for their comments. We have responded to each comments points by points and it is attached for the review of the editor.

Attachment Submitted filename: RESPONSE TO REVIEWERS COMMENTS.docx

# Peer review: Decision Letter 1

**DOI:** 10.1371/journal.pone.0358430.r004

**Authors:** Harikrishnan U

1 Sep 2026

Prevalence and Associated Factors of Sexually Transmitted Infections among Unmarried Undergraduate University Students

PONE-D-25-60693R1

Dear Dr. Hormenu,

We’re pleased to inform you that your manuscript has been judged scientifically suitable for publication and will be formally accepted for publication once it meets all outstanding technical requirements.

Within one week, you’ll receive an e-mail detailing the required amendments. When these have been addressed, you’ll receive a formal acceptance letter and your manuscript will be scheduled for publication.

An invoice will be generated when your article is formally accepted. Please note, if your institution has a publishing partnership with PLOS and your article meets the relevant criteria, all or part of your publication costs will be covered. Please make sure your user information is up-to-date by logging into Editorial Manager at Editorial Manager® and clicking the ‘Update My Information' link at the top of the page. For questions related to billing, please contact billing support.

If your institution or institutions have a press office, please notify them about your upcoming paper to help maximize its impact. If they’ll be preparing press materials, please inform our press team as soon as possible -- no later than 48 hours after receiving the formal acceptance. Your manuscript will remain under strict press embargo until 2 pm Eastern Time on the date of publication. For more information, please contact onepress@plos.org.

Kind regards,

Harikrishnan U, Ph.D

Academic Editor

PLOS One

Additional Editor Comments (optional):

Reviewers' comments:

Reviewer's Responses to Questions

**Comments to the Author**

Reviewer #1: All comments have been addressed

Reviewer #4: All comments have been addressed

**********

2. Is the manuscript technically sound, and do the data support the conclusions?

Reviewer #1: Yes

Reviewer #4: Yes

**********

3. Has the statistical analysis been performed appropriately and rigorously?

Reviewer #1: Yes

Reviewer #4: Yes

**********

4. Have the authors made all data underlying the findings in their manuscript fully available?

The PLOS Data policy

Reviewer #1: Yes

Reviewer #4: Yes

**********

5. Is the manuscript presented in an intelligible fashion and written in standard English?

Reviewer #1: Yes

Reviewer #4: Yes

**********

Reviewer #1: The authors have presented edited the abstract to improve clarity and objectivity. Judgmental statement in the background has been edited for neutrality. Other recommended edits are executed satisfactorily.

Reviewer #4: The article offers helpful details regarding the research population and intervention and is pertinent to the review topic. The methods are described in an understandable manner. The article seems appropriate for additional screening and full-text review.

**********

what does this mean?). If published, this will include your full peer review and any attached files.

If you choose “no”, your identity will remain anonymous but your review may still be made public.

**Do you want your identity to be public for this peer review?**  For information about this choice, including consent withdrawal, please see our Privacy Policy

Reviewer #1: No

Reviewer #4: No

**********

# Editor report: Acceptance letter

**DOI:** 10.1371/journal.pone.0358430.r005

**Authors:** Harikrishnan U

PONE-D-25-60693R1

PLOS One

Dear Dr. Hormenu,

I'm pleased to inform you that your manuscript has been deemed suitable for publication in PLOS One. Congratulations! Your manuscript is now being handed over to our production team.

At this stage, our production department will prepare your paper for publication. This includes ensuring the following:

* All references, tables, and figures are properly cited

* All relevant supporting information is included in the manuscript submission,

* There are no issues that prevent the paper from being properly typeset

You will receive further instructions from the production team, including instructions on how to review your proof when it is ready. Please keep in mind that we are working through a large volume of accepted articles, so please give us a few days to review your paper and let you know the next and final steps.

Lastly, if your institution or institutions have a press office, please let them know about your upcoming paper now to help maximize its impact. If they'll be preparing press materials, please inform our press team within the next 48 hours. Your manuscript will remain under strict press embargo until 2 pm Eastern Time on the date of publication. For more information, please contact onepress@plos.org.

You will receive an invoice from PLOS for your publication fee after your manuscript has reached the completed accept phase. If you receive an email requesting payment before acceptance or for any other service, this may be a phishing scheme. Learn how to identify phishing emails and protect your accounts at https://explore.plos.org/phishing.

If we can help with anything else, please email us at customercare@plos.org.

Thank you for submitting your work to PLOS One and supporting open access.

Kind regards,

PLOS One Editorial Office Staff

on behalf of

Dr. Harikrishnan U

Academic Editor

PLOS One
